# Overview of Caffeine Effects on Human Health and Emerging Delivery Strategies

**DOI:** 10.3390/ph16081067

**Published:** 2023-07-27

**Authors:** Sofia M. Saraiva, Telma A. Jacinto, Ana C. Gonçalves, Dário Gaspar, Luís R. Silva

**Affiliations:** 1 CPIRN-UDI/IPG, Center of Potential and Innovation of Natural Resources, Research Unit for Inland Development (UDI), Polytechnic Institute of Guarda, 6300-559 Guarda, Portugal; sofiasaraiva@ipg.pt (S.M.S.); telmajacinto@ipg.pt (T.A.J.); 2 CICS-UBI—Health Sciences Research Centre, University of Beira Interior, 6201-001 Covilhã, Portugal; anacarolinagoncalves@sapo.pt; 3 Department of Sport Sciences, University of Beira Interior, 6201-001 Covilhã, Portugal; dariogaspar7@gmail.com; 4 Department of Chemical Engineering, University of Coimbra, CIEPQPF, Rua Sílvio Lima, Pólo II—Pinhal de Marrocos, 3030-790 Coimbra, Portugal

**Keywords:** caffeine, health benefits, athletic effects, dietary supplements, nanocarriers

## Abstract

Caffeine is a naturally occurring alkaloid found in various plants. It acts as a stimulant, antioxidant, anti-inflammatory, and even an aid in pain management, and is found in several over-the-counter medications. This naturally derived bioactive compound is the best-known ingredient in coffee and other beverages, such as tea, soft drinks, and energy drinks, and is widely consumed worldwide. Therefore, it is extremely important to research the effects of this substance on the human body. With this in mind, caffeine and its derivatives have been extensively studied to evaluate its ability to prevent diseases and exert anti-aging and neuroprotective effects. This review is intended to provide an overview of caffeine’s effects on cancer and cardiovascular, immunological, inflammatory, and neurological diseases, among others. The heavily researched area of caffeine in sports will also be discussed. Finally, recent advances in the development of novel nanocarrier-based formulations, to enhance the bioavailability of caffeine and its beneficial effects will be discussed.

## 1. Introduction

Currently, special attention is being paid to natural molecules and their putative therapeutic effects to delay, or even prevent, the occurrence of many diseases and improve the health status of the population [1]. Indeed, their ingestion is widely believed to have fewer or no adverse effects on humans than most synthetic molecules, and they are also cheaper and easier to obtain [2,3,4]. Caffeine, in particular, has been the subject of intense and in-depth research on the human organism regarding its health-promoting effects and possible beneficial effects on the performance of athletes, especially through its ability to improve anaerobic and aerobic performance, muscle efficiency, and speed, and to reduce fatigue [5,6,7,8,9]. Caffeine is probably the most commonly ingested psychoactive substance in the world, found mainly in coffee, soft drinks, tea, cocoa and chocolate-like products, yerba matte leaves, guarana berries, and some pharmaceuticals [10]. It is rapidly absorbed and distributed in all human tissues, reaching maximum plasma concentrations 30–120 min after oral intake [9].

As far as we know, in vivo studies have already reported that caffeine stimulates the central nervous system by acting as an antagonist of A1 and A2 adenosine receptors, promotes adrenaline release, increases dopamine, noradrenalin, and glutamate levels, blood circulation and respiratory rate, mobilizes intracellular calcium stores, and alters fat and carbohydrates metabolism in the human body by stimulating lipolysis, thanks to its ability to inhibit phosphodiesterase enzymes [11,12,13,14,15,16]. In addition, caffeine also increases energy, alertness, excitement, and mood [17,18]. According to the European Food Safety Authority (EFSA), a habitual daily consumption of caffeine up to 400 mg (5.7 mg/kg bw per day for a 70 kg adult) by adults, and 200 mg by pregnant or breastfeeding women is considered to be safe [19]. Exceeding this dose or the sudden cessation of caffeine intake may cause anxiety, insomnia, hallucinations, hypertension and headache, gastrointestinal and sleep disturbances, diuresis, dehydration, tremors, palpitations, and cardiac arrhythmias given the stimulant effects of caffeine [20,21,22,23]. Regarding children and adolescents, the EFSA considers that there is insufficient information [19]. Considering caffeine’s high consumption, as well as its increasing commercial availability in pure form, and presence at high concentrations in products such as dietary supplements, the US Food and Drug Administration (FDA) and EFSA warn about the risks of its consumption at high doses [24,25].

Nonetheless, considering the different beneficial biological and physiological effects of caffeine, extensive studies have been conducted to determine its full health potential. In this line, caffeine encapsulation, alone or combined with other molecules, has been performed to increase its biological activities [26,27,28,29,30]. Therefore, the main objective of this review is to discuss the biological potential of caffeine for human health, highlighting its anticancer, immunological, anti-inflammatory, cardiovascular, and neurological protective effects, as well as its effects on the performance of athletes.

## 2. Chemical Structure and Main Natural Sources of Caffeine

**Figure 1:**
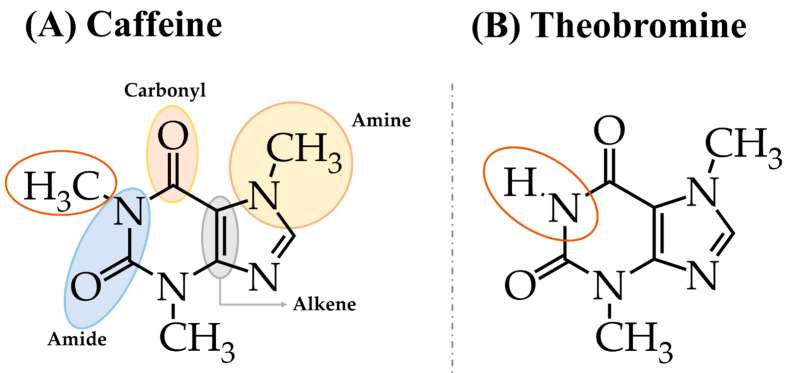
Chemical structure and functional groups of caffeine (**A**) and theobromine (**B**).

Caffeine (C_8_H_10_N_4_O_2_; Figure 1A), also known as 1,3,7-trimethylxanthine, belongs to the group of methylxanthines, which are alkaloids [11,31]. Together with theobromine, its precursor (Figure 1B), both are synthetized by the fruits, leaves, and seeds of many plants and trees to protect them from diseases and predators [22,32,33].

Regarding their structure, both compounds are carbon- and nitrogen-based molecules composed of two purine rings, a pyrimidine ring (C5-ring), and an imidazole ring (C6-ring), both of which have two nitrogen atoms [34,35]. Their functional groups are an amide (a carbonyl group bonded to carbon and nitrogen atoms), an amine (at least one hydrocarbon group bonded to a nitrogen atom), and an alkene (an unsaturated hydrocarbon with a double bond between two carbon atoms) [34]. Caffeine serves as a hydrogen bond acceptor because three of its four nitrogen atoms are methylated [36].

Although caffeine and theobromine share similarities at the physical and chemical levels, caffeine has an additional methylene group and exerts stronger central nervous system effects than theobromine [37,38].

Regarding caffeine consumption, recent statistical data show that more than 85% of American adults consume caffeine daily (135 mg per day) [39,40]. In Europe, a higher caffeine consumption is observed (values ranging from 37 to 319 mg per day), especially in the Netherlands (411 mg per day), Denmark (390 mg per day), Finland (329 mg per day), Austria (300 mg per day), and Switzerland (288 mg per day) [19,41]. Compared to other non-European countries, Brazil and Argentina also have a high consumption of caffeine (mean values of 40 and 100 mg per day, respectively), as well as Australia (232 mg per day) and Japan (169 mg per day) [41]. In contrast, lower amounts of caffeine are consumed in China (16 mg per day), Angola (4 mg per day), and Kenya (50 mg per day) [41].

The main sources of caffeine are coffee and tea, energy beverages, sodas and soft drinks, and dark chocolate (see Table 1) [31,41,42,43,44]. As expected, coffee and chocolate are the most popular sources of caffeine worldwide [31,42]. Among the different types of coffee (beverage), American coffee is the most caffeinated (91.7–213.3 mg per 100.0 mL) [42], followed by Scotland espresso (66.0–276.0 mg per 13.0–90.0 mL) [31]. As for tea, black tea and Yerba Mate contain considerable amounts of this molecule (both around 40.0 mg per 236.0 mL) [41]. Among soft drinks, Mountain Dew Rise and Diet Coke have considerable amounts of caffeine (180.0 mg per 473.0 mL and 46.0 mg for 354.0 mL, respectively) [41]. Among energy drinks, Java Monster 300 and Rockstar XDurance present the highest caffeine contents (amounts around 300.0 mg per 443.0 and 473.0 mL, respectively), followed by Full Throttle (160.0 mg per 473.0 mL) [41]. In addition, as expected, energy shots, such as Spike Energy Double Shot and Bang Shot, are also rich in caffeine (levels of 350.0 and 300.0 mg per 125.0 and 88.0 mL, respectively) [41]. Finally, Cran Energy juice and Water Joe also have significant amounts of caffeine (70.0 mg per 295.0 and 591.0 mL, respectively) [41,45]. The presence of caffeine is also reported in dark chocolate (8.0 mg per 1.0 g) [41].

## 3. Benefits of Caffeine on Health

For our search, we used Web of Science. The search restrictions were based on language (English), year of publication from 2018 to present, and type of publication set to journal. The keywords used for the search were “caffeine” in combination with any of these other keywords: “cancer”, “anticancer”, “antitumor”, “anti-tumor”, anti-cancer”, “neurodegenerative diseases”, “autoimmune diseases”, “immunological”, “immunomodulatory”, “immune system”, “anti-inflammatory”, and “cardiovascular”. In the following subsections, we provide an overview of the latest research regarding the impact of caffeine in different illnesses such as cancer, autoimmune diseases, immunomodulation, and ocular, respiratory, neurodegenerative, and cardiovascular diseases. 

### 3.1. Cancer

Cancer is one of the leading causes of death worldwide. It was estimated that in 2020, there were 19.3 million cancer cases, which resulted in 10.0 million cancer deaths [46,47]. By 2030, it is estimated that over 22 million people will develop cancer [47,48]. In addition, cancer is responsible for a significant economic burden on both the health care system and patients [48]. 

As early as 2000, Hanahan and Weinberg defined the key features (i.e., “hallmarks of cancer”) that describe the characteristics necessary to promote cancer growth and metastasis. These hallmarks are self-sufficiency in growth signals, insensitivity to antiproliferative signals, resistance to apoptosis, limitless replicative potential, the induction of angiogenesis, and the activation of tissue invasion and metastasis [49]. In 2011, the authors revised the original hallmarks and added two more cancer-promoting features (genomic instability and tumor-promoting inflammation) and two more hallmarks (deregulation of cellular energetics and avoidance of immune destruction) [50]. As the understanding of cancer underlying mechanisms of progression has grown, as have the available experimental and computational tools; early in 2022, Hanahan reviewed the previously discussed features and included new additional features of cancer, namely, (i) phenotypic plasticity, (ii) non mutational epigenic reprogramming, (iii) polymorphic microbiomes, and (iv) senescent cells [51].

The role of coffee components in suppressing some of the cancer hallmarks defined by Hanahan and Weinberg [52,53] has been reviewed by Gaascht et al. and Cadóna et al., while other authors have fully elucidated the effect of caffeine on the cell cycle [54]. Caffeine anticancer activity has been widely studied [55], and the below-stated findings demonstrate the capacity of caffeine to overcome some of the cancer-promoting hallmarks, such as resistance to cell death and cellular senescence, that play an important role in cancer progression [51]. Further, several works state that caffeine may induce apoptosis through numerous pathways, such as p-53-dependent and -independent, phosphatase and tensin homolog, PI3K/protein kinase B (AKT), and mammalian target of rapamycin (mTOR) pathways [56].

El Far et al. studied the effect of caffeine and other natural substances on the senescent cells of colon and breast cancers. After inducing senescence with doxorubicin, the cells were treated with various doses of caffeine (0, 5, 10, 15, 20, 30, 40, 50, and 60 mM). The IC_50_ of caffeine against doxorubicin-treated HCT116 and MCF7 cells was 13.36 ± 2.29 mM and 17.67 ± 3.98 mM, respectively. The authors also examined caffeine-induced apoptosis in both senescent and proliferative cells. At concentrations of 10 and 15 mM, caffeine induced a significant increase in apoptosis in senescent HTC116 cells, and at concentrations of 5, 10, and 15 mM in senescent MCF7 cells compared with proliferative cells [56]. In another study, Machado et al. evaluated the effect of caffeine on two breast cancer cell lines (MCF-7 and MDA-MB-231). The results showed that caffeine at a concentration of 2.5 mM and 5 mM for MCF-7 and MDA-MB-231, respectively, reduced cell viability and induced apoptosis [57]. The antitumoral effects of caffeine were studied in diverse cancer in vitro models, such as glioblastoma, melanoma, and pancreatic and lung cancers [58,59,60]. 

The antitumoral effects of caffeine have also been evaluated in in vivo tumor models. Venkata Charan Tej and collaborators investigated the effect of caffeine on the carcinogen-induced tumor model of fibrosarcoma. After 250 days of 3-MCA inoculation, there was a dose-dependent decrease in the tumor incidence and growth rate in the groups treated with caffeine (1.030, 2.060, and 4.120 mM) [61]. The anti-tumoral effect of caffeine was related to its action on cytotoxic T lymphocytes. On one hand, caffeine led to a higher percentage of cytotoxic T cells in the tumor, and on the other hand, it decreased the expression of programmed cell death protein 1 (PD-1) on these cells. In addition, it also increased the levels of pro-inflammatory cytokines such as TNF-α and IFN-γ. These results are in line with the previously known inhibitory effect of caffeine on the adenosine-A2a receptor pathway [62], which is one of the immunosuppressive pathways involved in cancer [63,64]. This capacity of caffeine to modulate the immune system in the tumor surroundings alters another important hallmark (i.e., the ability to avoid immune destruction). The modulation of the PD-1, an important immune checkpoint, and consequent enhancement of the T cell responses can exert an antitumor effect. In fact, the inhibitors of this protein are one of the immunotherapies approved by the FDA [65]. 

The therapeutic effect of caffeine was also demonstrated for renal carcinoma. Xu et al. showed, through in silico studies, that caffeine is able to bind to glucose-6-phosphate dehydrogenase (G6PDH), which is considered a biomarker and potential therapeutic target for this type of cancer. Consistent with the above results, in this study, the use of caffeine at concentrations of up to 0.016 mM for in vitro studies and 60 and 120 mg/kg/day for in vivo studies decreased the viability and proliferation of ACHN and 786-O cancer cells both in vitro and in vivo [64]. G6PDH is an important target in cancer given that is normally upregulated in different cancers and its dysregulation can provide valuable conditions for cancer progression [66]. Further, it also has an important role in maintaining the redox balance and biosynthesis of nucleotides and lipids, which is part of another cancer hallmark (i.e., reprogramming cellular metabolism) [67].

As previously mentioned, caffeine has also been tested in combination with other drugs in order to potentiate the antitumoral effect [68,69,70,71]. Higuchi et al. evaluated the efficacy of oral recombinant methioninase (o-rMETase) in combination with caffeine and doxorubicin in an orthotopic xenograft mouse model of synovial sarcoma. After two weeks of treatment, the group treated with the combinatorial treatment was able to induce tumor regression. According to the authors, this can be explained by the ability of caffeine to induce mitotic catastrophe [72]. Other examples of caffeine combination with different drugs are depicted in Table 2. 

Understanding the effects of caffeine on cancer and the mechanisms underlying this effect is of extreme importance. Table 2 summarizes the most recent (from 2018) works on this topic. These studies also contribute to determining the necessary caffeine quantities to achieve a therapeutic effect and to ensure the safe use of caffeine.

### 3.2. Anti-Inflammatory and Immunomodulation

#### 3.2.1. Autoimmune Diseases and Immunomodulation

Inflammation is usually caused by infection or damage to a tissue [84]. Caffeine has the ability to exert modulation on the immune system. The immune response can be divided into two types: (i) innate and (ii) adaptive immunity [85]. Acute inflammation is a mechanism of innate immunity, whereas chronic inflammation usually contributes to the development of various diseases, such as metabolic disorders, neurodegenerative diseases, and even cancers [86,87]. The effect of caffeine on the innate immune system is related to the reduction in macrophage, neutrophil, and monocyte chemotaxis [88,89]. As for adaptive immunity, the effect of caffeine is due to the inhibition of Th1 and Th2 cell proliferation, as well as to the alteration of B cell function and the consequent reduction in antibody production [89,90,91,92]. Several authors, such as Horrigan et al., Açıkalın et al., and Al Reef et al., already reviewed, in depth, the impact of caffeine on the immune system and its capacity to alleviate autoimmune diseases [88,93,94].

Considering the immunomodulatory effects of caffeine, Wang et al. evaluated its effects on multiple sclerosis. Experimental autoimmune encephalomyelitis is the standard animal model for multiple sclerosis. After inducing the disease in C57BL/6 mice, these were treated with caffeine (10, 20, or 30 mg/kg/day) in drinking water. The results showed that caffeine could reduce inflammatory cell infiltration, the degree of demyelination, and microglial in vivo. It also reduced NLRP3 and p62 protein levels. In vitro assays indicated that caffeine promoted autophagy [95]. In another study, Ghaffary et al. evaluated the potential of mesenchymal stem cells to reduce the severity of rheumatoid arthritis. Wistar rats were treated with mesenchymal stem cells that had previously been incubated with various concentrations of caffeine. The results showed that the rats treated with mesenchymal stem cells, previously treated with 0.5 mM of caffeine, presented decreased disease severity and serum levels of C-reactive protein, nitric oxide, myeloperoxidase, and TNF-α. In addition, the IL-10 serum levels and the weight of the treated rats increased [96].

#### 3.2.2. Ocular Diseases

Adenosine receptors are also expressed by retinal endothelial and retinal pigment epithelial (RPE) cells, as well as choroid and choroidal cells [97]. Therefore, caffeine may also have beneficial effects in ocular diseases, such as choroidal neovascularization and retinal inflammation. 

Retinal inflammation is involved in ocular diseases as age-related macular degeneration (AMD) and diabetic retinopathy (DR), among others. For example, AMD is characterized by elevated vitreous levels of IL-1*β* [98] and plasmatic tumor necrosis receptor 2 (TNF-R2) and low levels of brain-derived neurotrophic factor (BDNF) in the aqueous humor, which negatively affect photoreceptor and retinal ganglion cells’ survival [99]. Conti et al. demonstrated that caffeine has an anti-inflammatory effect in RPE cells, decreasing the expression of IL-1*β*, IL-6, and TNF-*α*, as well as the nuclear translocation of nuclear factor kappa B (NF-*κ*B). In addition, the topical instillation of caffeine in an ischemia-reperfusion injury mice model was shown to restore physiological BDNF levels and reduce the mRNA levels of IL-6 in the retina, demonstrating its potential for the treatment of retinal inflammation and degeneration [100]. The effect of caffeine on choroidal adenosine receptors, the reduction in cell migration to the injured area, and angiogenesis demonstrate the importance of caffeine in attenuating choroidal neovascularization [97]. Despite the potential of caffeine in the management of such ocular conditions, the available studies are still scarce.

#### 3.2.3. Respiratory Diseases

Currently, there are respiratory diseases for which caffeine is used as a clinical treatment, namely, premature infant diseases such as apnea and bronchopulmonary dysplasia (BPD). BPD is a common neonatal pulmonary complication with a prevalence of 45% in preterm infants [101]. BPD is associated with a nonspecific inflammatory response involving the activation of Toll-like receptors (TLRs), NOD-like receptors (NLRs), and increased levels of pro-inflammatory cytokines (IL-1*β*, IL-6, IL-8, IL-18, TNF*α*) [102]. In addition, NLR3 (NOD-, LRR-, and pyrin domain-containing protein 3), a key player in the pathogenesis of BPD, is responsible for the release of pro-inflammatory cytokines (IL-1*β* and IL-18) and alveolar cell death through various mechanisms [103,104]. Caffeine is the most commonly used medication for extreme prematurity (less than 28 weeks) and is also very commonly prescribed for very early preterm birth (28 to 32 weeks) [105]. As clinically shown, the early initiation of caffeine treatment (5 and 10 mg/kg/day) is important to achieve a successful outcome. Early treatment significantly reduced BPD incidence and mortality in low-birth-weight neonates [106]. Despite the use of caffeine and its clear benefits, the mechanisms behind the clinical benefits in these diseases are not fully understood.

In vitro studies showed that the treatment of lipopolysaccharide (LPS)-induced macrophages with caffeine caused a reduction in caspase-1 expression and the inhibition of the NLRP3 inflammasome, demonstrating its potential effect on this important target. Moreover, in vivo, the treatment of newborn mice with hypoxia-induced lung injury with caffeine was shown to significantly increase A2a receptor expression and inhibit the NLRP3 inflammasome protein and NF-*κ*B pathway in the lung. The effect of caffeine on these key regulators attenuated inflammatory infiltration, reduced oxidative stress, decreased alveolar cell death, and promoted alveolar development [107]. Similar results were also observed in another study; specifically, caffeine caused a decrease in NF-*κ*B and pro-inflammatory factor levels, increased the expression of A1, A2a, and A2b receptors, and decreased cell death in the lung [108].

Table 3 summarizes recent research findings on the anti-inflammatory effects of caffeine and its effects on autoimmune diseases.

### 3.3. Neurodegenerative Diseases

By 2050, the number of dementia cases worldwide is estimated to be 36.5 million [127]. There are several neurodegenerative diseases, such as Parkinson’s disease, Alzheimer’s disease, Huntington’s disease, and multiple sclerosis [128,129]. For example, Parkinson’s disease is triggered by the loss of neurons, which leads to a decrease in dopamine levels. In Alzheimer’s disease, there is a deposition of extracellular deposits of amyloid-beta peptides and neurofibrillary tangles [130,131]. 

Caffeine is considered the most widely consumed psychoactive stimulant in the world. This natural compound is able to cross the blood–brain barrier [132,133] and, according to the literature, may exert a stimulant effect on the central nervous system by modulating several molecular targets, such as the (i) antagonism of adenosine receptors, (ii) promotion of intracellular calcium mobilization, (iii) inhibition of phosphodiesterase, and (iv) inhibition of GABA_A_ receptors. However, except for the blockade of adenosine receptors and consequent inhibition of neurotransmitter-induced signaling pathways, the other mechanisms only exert their effects at toxic concentrations of caffeine [132,134,135,136]. Recently, Ruggiero et al. reviewed the available literature on the protective effects of caffeine in various neurodegenerative diseases [137]. Among these studies, some emphasized the neuroprotective role of caffeine. For example, Manolo et al. showed that caffeine, at a concentration of 10 mM, is able to protect 96% of the dopaminergic neurons. The co-administration of olanzapine and caffeine did not result in neuroprotection, implying that both dopamine D2-like and A2a receptors are required for neuroprotection [138]. In an in silico study of Parkinson’s disease, the authors demonstrated that caffeine has the ability to bind to both wild-type and mutant parkin protein [139]. The mutation of parkin protein is the most common cause of Parkinson’s disease, as is the abnormal secretion and accumulation of α-synuclein [140,141]. This last part was detected in the following in vivo studies. Luan et al. investigated whether caffeine could protect against mutant α-synuclein-induced toxicity. Exposing mice to 1 g/L of caffeine in drinking water attenuated apoptotic neuronal cell death as well as microglia and astroglia reactivation, culminating in synucleinopathy [142]. In a similar study, Yan et al. investigated synergetic neuroprotection between caffeine and eicosanoyl-5-hydroxytryptamide. Both compounds are present in coffee and showed no effect at subtherapeutic doses, whereas their combination reduced the accumulation of phosphorylated α-synuclein, and maintained neuronal integrity and function [143]. Table 4 summarizes the most recent research on the neuroprotective effects of caffeine in neurodegenerative diseases and other conditions.

### 3.4. Cardiovascular Diseases

Cardiovascular disease (CVD), the leading cause of mortality, accounted for 17.8 million deaths worldwide between 1980 and 2017 [160]. By 2030, an estimated 23.6 million people per year will die due to CVD. Caffeine intake, particularly through the consumption of coffee, tea, and other products, has shown various cardiovascular effects. Turnbull et al. reviewed more than 300 studies regarding the effects of caffeine on cardiovascular health, published from the late 1980s to 2017. Overall, the results suggest that caffeine consumption does not increase the risk of CVD and may have a protective effect against this group of diseases [161]. However, recent studies on this topic have shown that high caffeine consumption may have the opposite effect.

A study of 347,077 people (UK Biobank) concluded that coffee consumption may modestly increase the risk of cardiovascular disease. A nonlinear association was found between long-term coffee consumption and cardiovascular disease. Individuals who consumed coffee in high doses (>6 cups/day, >450 mg caffeine/day) were more likely to develop cardiovascular disease (22%) than those who consumed less coffee (1–2 cups/day or 75–150 mg caffeine/day) [162]. In addition, the authors examined the association between coffee consumption, plasma lipids, and CVD risk in 362,571 individuals (UK Biobank). The results showed that high coffee consumption (>6 cups/day) may increase CVD risk by increasing the levels of low-density lipoprotein cholesterol (LDL-C), total cholesterol (total-C), and apolipoprotein B (ApoB) [163]. 

However, other studies have reported the potential beneficial effects of moderate coffee consumption, in line with Turnbull et al.’s literature review [161]. For instance, a study involving 20,487 Italian participants concluded that moderate coffee consumption (3–4 cups/day) was associated with a low risk of CVD-related mortality. In addition, an inverse correlation was found between NT-proBNP levels (N-terminal fragment of the B-type natriuretic peptide, which is associated with higher stroke risk) and coffee consumption [164]. Similarly, a study of more than 500,000 participants in England reported that a caffeine intake of 121–182 mg/day from coffee (2–3 cups/day) or tea (4–6 cups/day) was associated with a low risk of coronary artery disease [165]. In addition, a US follow-up study of 23,878 participants over 16 years found that the daily caffeine consumption of about 100–200 mg or >200 mg is associated with a lower risk of CVD mortality [166]. An inverse association between coffee consumption and CVD risk factors (blood pressure and arterial stiffness) was also observed in another study, showing the beneficial effect of moderate coffee consumption [167]. A similar association was observed concerning coffee consumption and hypertension risk [168].

Therefore, despite some studies linking high coffee or caffeine consumption to CVD risk, most studies have reported that its moderate consumption has potentially beneficial and even protective effects on CVD. Table 5 summarizes the recent research on the effects of caffeine on CVD.

## 4. Caffeine Impact on Sports Performance

Coffee’s best-known constituent, caffeine, is the most widely consumed psychotropic drug in the world, with an estimated daily intake of up to 4 mg/kg body weight in American adults [173,174,175,176]. It is a psychostimulant that can lead to physical dependence [177]. Caffeine intake is widespread among inactive individuals and high-performance athletes, especially since 2004, when it was removed from the World Anti-Doping Agency’s list of banned substances for competition [178]. It is also readily available in various forms such as capsules, powders, caffeinated beverages, and energy drinks [173].

However, while there is evidence that caffeine improves athletic performance [173,174,175,176,177,178,179,180,181], due to particular protocols and study designs, some research seems conflicting. Some studies show ergogenic effects on aerobic endurance (>90 min), high-intensity efforts (20–60 min), muscular endurance, sprint performance and maximal strength (0 to 5 min), and ultra-endurance (>240 min) and endurance races with prolonged intermittent sprints (team sports), while others report no evidence for its administration [180,181,182]. We assume that an ergogenic substance is a substance used with the aim of improving athletic performance and promoting recovery after exercise by delaying fatigue. The word is of Greek origin: ergo (work) and gen (generation). As a result, it is commonly consumed by athletes, and research suggests that 75 to 90% of athletes consume caffeine before or during athletic competition [181]. In an analysis of 20,686 urine samples from elite athletes, 73.8% of the samples contained caffeine at concentrations greater than 0.1 µg/mL, suggesting that three out of four athletes consume caffeine before or during competition [175].

It should be recalled that the consumption of caffeine is not prohibited for athletes, with the maximum allowable concentration being 12 mg/L of urine (International Olympic Committee). The fact that caffeine affects the nervous system, adipose tissue, and skeletal muscle originally led to the hypothesis that caffeine might affect athletic performance. For example, caffeine may increase skeletal muscle contractile force at submaximal contraction and increase the athlete’s pain threshold or perceived exertion, which could lead to longer training sessions [180,181].

However, it should be remembered that caffeine intake has several side effects. Blood pressure increases both at rest and during exercise and heart rate increases, and it may impair recovery and sleep patterns, most likely in athletes who do not regularly consume caffeine [180]. In addition, Martins et al. demonstrated that high doses of caffeine have side effects. In a recent study using a caffeine dose of 12 mg/kg, almost all participants reported side effects such as tachycardia and palpitations, anxiety or nervousness, headache, and insomnia [175].

However, according to our research, it seems important to us to better evaluate certain aspects to achieve better scientific clarification with implications for practice, such as the ideal dosage, time of intake, abstinence, training time vs. caffeine consumption, physiological factors, gender, and caffeine users or not.

### 4.1. Optimal Dosage

Higher-than-ideal caffeine doses, 3–6 mg/kg, before exercise do not further improve athletic performance. Additional and higher doses of caffeine may lead to side effects in athletes [180].

Low doses of caffeine (~200 mg) have also been shown to improve attention, alertness, and mood, and cognitive processes during and after strenuous exercise. Thus, the ergogenic effects of low doses of caffeine appear to be due to changes in the central nervous system [180].

The generally accepted dosage of caffeine for performance enhancement is between 3 and 6 mg/kg, 60 min before exercise [175].

Although a meta-analysis reported that caffeine intake can be ergogenic in a variety of physical activities, the “optimal” caffeine dose remains difficult to determine [178].

### 4.2. Timing of Intake

The early ingestion of caffeine prior to physical activity has been shown to enhance performance. For example, caffeine can improve performance during high-intensity sprints when taken 45–60 min before exercise [176].

Because caffeine has so many positive effects on exercise performance, it can—and perhaps should—be taken before or during exercises. For most sports, it is recommended that caffeine be taken about 60 min before the start of the first set of the training session if used before exercise. This period varies depending on the individual, the type of event, and the type of caffeine ingested, with caffeinated mouthwashes and chewing gums generally requiring much less time. For longer training sessions, there is evidence that ingesting caffeine later in the day, and at lower doses, may be effective [181]. Other interesting data refer to the concentration peak that occurs in the first 15 min [183].

The isolated consumption of anhydrous caffeine results in maximal plasma peaks of the substance between 30 and 90 min after the consumption of low (2–3 mg/kg), moderate (3–6 mg/kg), or high doses (6–9 mg/kg) [175,184].

### 4.3. Abstinence

It appears that, short-term, caffeine withdrawal before competitions does not enhance the ergogenic effects of caffeine in habitual users. Withdrawal is associated with numerous negative consequences, including headache, fatigue, irritability, muscle pain, sleep disturbance, and nausea. However, these acute withdrawal symptoms, shortly before important competitions, may have a negative impact on the subjective self-confidence and well-being of the athlete [181].

### 4.4. Training Time vs. Caffeine Consumption

Increases in physical performance as a function of training time have been demonstrated in various sports. Studies suggest that anaerobic and aerobic activities may be more powerful due to the diurnal fluctuations of the circadian cycle between 4 and 8 pm. Morning caffeine consumption had a more beneficial effect than afternoon consumption [175].

### 4.5. Physiological Factors

Hypothetically, the potential performance enhancement from caffeine ingestion may be greater in trained individuals than in untrained individuals because trained individuals have an enhanced neuromuscular action potential. Trained individuals have a higher concentration of adenosine A2a receptors than untrained individuals [175,185].

The main finding of this review is that very low doses of caffeine (>1–2 mg/kg, generally taken 60 min before exercise) improve resistance training performance in terms of muscle strength, muscle endurance, and average speed [174].

Aerobic endurance appears to be the sport in which caffeine consumption most consistently produces moderate to significant benefits, although the magnitude of the effect varies among individuals [185].

### 4.6. Gender

Caffeine ingestion positively affects resistance exercise performance in women, and the magnitude of these effects appears to be comparable to those observed in men [184]. Even considering the woman’s menstrual cycle, a study showed that caffeine increased peak aerobic cycling power in the early follicular, preovulatory, and mid-luteal phases. Thus, the ingestion of 3 mg of caffeine per kg of body mass might be considered an ergogenic aid for eumenorrheic women during all three phases of the menstrual cycle [186].

### 4.7. Caffeine Consumers or Not

For the first study using a performance test, 17 moderately trained men were recruited, 8 of whom did not routinely consume caffeine (<25 mg/day) and 9 of whom regularly consumed caffeine (>300 mg/day). It was found that there were no differences between the groups in time to exhaustion at any of the doses, suggesting that habitual caffeine consumption does not attenuate the ergogenic effects of caffeine [181]. In another study, on cycling, habitual caffeine intake was found to have no effect on athletic performance, suggesting that habituation to caffeine has no negative effect on caffeine ergogenesis [187].

## 5. Future Directions: Nanotechnology-Based Delivery Strategies

Caffeine is usually consumed through the ingestion of beverages, especially coffee, tea, and pharmaceuticals, which allows for rapid absorption and distribution in all tissues [9]. However, caffeine has a short half-life (3–5 h) [188]. In addition, the oral intake of high concentrations of caffeine may cause gastrointestinal problems [189] and its wide distribution may lead to undesirable side effects, such as the stimulation of the nervous system. 

**Figure 2:**
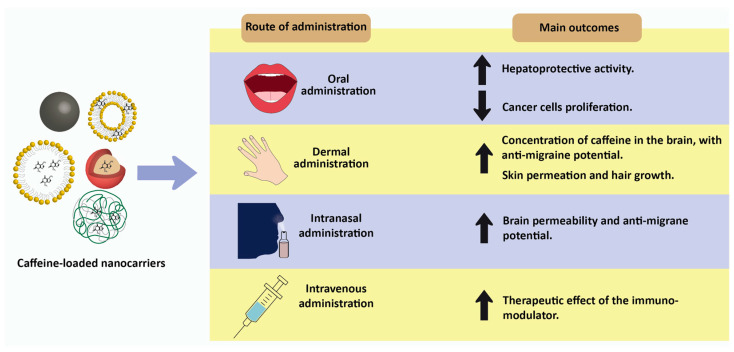
Possible administration routes for caffein-loaded nanosystems and their main outcomes.

Nanotechnology is a multidisciplinary field that enables the manipulation of matters at the nanoscale (1 to 100 nm) and the creation of novel devices with unique properties [190]. Nanotechnology is frequently explored for drug delivery to a target tissue. Drug delivery systems (DDS) or nanocarriers offer important advantages for caffeine delivery, namely, a high loading capacity, the co-encapsulation of different drugs, controlled and sustained release, a high surface area allowing greater interaction with tissue, and a high ability to permeate through tissues [191]. In addition, other routes of administration besides oral can be used, such as intranasal [192] and dermal [188] (Figure 2)**.**

Nanocarriers’ compositions are tailored depending on the drug(s), route of administration, and target tissue. Therefore, different nanocarrier compositions based on lipids, polymers, or metals have been proposed for caffeine delivery, as reported in this section. 

Lipidic nanocarriers have been widely explored for topical drug delivery through the skin for cosmetic and pharmaceutical applications [193]. The composition of lipid carriers is an important factor to be considered to improve skin permeation and therapeutic effects. For example, among the various phospholipids (1,2-distearoyl-snglycero-3-phosphocholine (DSPC), 1,2-dipalmitoyl-sn-glycero-3-phosphoglycerol, sodium salt (DPPG), 1,2-dilauroyl-sn-glycero-3-phosphocholine (DLPC), and 1,2-dimyristoyl-sn-glycero-3-phosphocholine (DMPC)) tested for liposome preparation and the topical delivery of caffeine, DPPG was the most promising. Ex vivo studies showed that DPPG was able to enhance the permeation of encapsulated and free caffeine through hairless rat skin by disrupting the lipid barrier of the stratum corneum (SC) [194]. A similar effect was observed for lipid nanocapsules (NCs) in porcine skin. The ability of lipid NCs to increase skin permeation of free caffeine has been attributed to the combination of several factors, namely, the occlusion effect of nanoparticles on the skin surface, accumulation in hair follicles, and the effect on barrier function of SC [195]. On the other hand, the incorporation of propylene glycol into phosphatidyl liposomes has been shown to enhance the permeation of caffeine through the skin, as demonstrated ex vivo in human full-thickness skin [196]. In this sense, the researchers proposed the combination of the lipolytic activity of caffeine with the increased permeation capacity of propylene glycol liposomes as a noninvasive treatment for cellulitis [196]. Amasya et al. also proposed semisolid lipid nanoparticles as a promising treatment for cellulitis because they can penetrate the skin and reach the adipose tissue [197].

Flexible liposomes composed of phosphatidylcholine and higher surfactant content (polysorbate 80 and polysorbate 20) were also proposed for the treatment of alopecia by topical application [198]. The therapeutic potential of caffeine in alopecia is due to its ability to inhibit 5-α-reductase and phosphodiesterase and increase vasodilatation and blood supply to hair follicles [199]. The nanocarriers co-encapsulating minoxidil and caffeine resulted in an increase in hair length comparable to the aqueous solution of the drugs and the commercial alcoholic solution. Nevertheless, liposomes loaded with caffeine and minoxidil led to a significant increase in hair weight, an indicator of healthy and strong hair, demonstrating the potential of liposomes for the treatment of hair loss [198]. Other types of nanosystems, namely, nanoemulsions containing eucalyptol and oleic acid, have been shown to accumulate in hair follicles and increase caffeine retention in these structures, demonstrating the potential of these nanosystems for the treatment of alopecia [200]. Considering that hair follicles are nourished by blood vessels, targeted accumulation in these structures may enhance the permeation of caffeine. Therefore, these approaches can also be used to develop novel therapeutics for diseases of other tissues to avoid systemic or oral delivery.

In this sense, proniosomes have been proposed for the treatment of migraine by the topical application of caffeine. As expected, caffeine-loaded proniosomes, applied topically to Swiss albino mice, were able to penetrate the skin. Moreover, the treatment resulted in a significantly higher caffeine concentration in the blood and brain, as well as prolonged and sustained effects, compared with orally administered caffeine solution [188]. Recently, the co-delivery of caffeine and ergotamine to the brain by intranasal administration (olfactory route) has also been proposed. Hybrid lipid–polymer nanoparticles of lecithin, poly(lactic-co-glycolic acid) (PLGA), and 1,2-dipalmitoyl-sn-glycero-3-phosphocholine functionalized with polyethylene glycol (PEGylated DPPC) showed a high encapsulation efficiency (87%) and controlled release over a period of 48 h. In addition, the results showed that the nanoparticles had high targeting accuracy in the brain without causing toxic effects. Furthermore, the synergistic effects of the drugs enhanced the anti-migraine effect [192].

The anti-cancer effect of caffeine has also been enhanced by the use of nanocarriers. Liu et al. prepared lipid-based nanosystems for the co-delivery of caffeine and imiquimod. Caffeine enhanced the therapeutic effect of the immunomodulator imiquimod and radiotherapy in an orthotopic breast cancer model. The authors suggested that this may be due to the modulation of the tumor microenvironment [201]. On the other hand, polymer-based nanocarriers, i.e., gelatin nanoparticles loaded with caffeine, showed the ability to decrease the viability and proliferative capacity of murine melanoma cells (B16F10) without causing significant cytotoxic effects in normal fibroblast cells (L929) [202]. Other studies have reported the combination of caffeine with metallic nanocarriers. For example, silver–caffeine complexes anchored to magnetic nanoparticles were proposed for the treatment of hepatocellular carcinoma [203]. This type of cancer is known to be resistant to radiotherapy and chemotherapy and can be caused by hepatitis-related infections. The most promising nanoparticles showed higher cytotoxicity against the cancer cells (hepatocellular carcinoma cells, HCC) than against the normal cells (normal hepatic cells, WRL-68). On the other hand, the targeted hyperthermia effect of the magnetic nanoparticles can improve the anti-tumor effect of the formulation and avoid the side effects of the commonly used therapeutics. In addition, these silver–caffeine magnetic nanoparticles also showed antibacterial activity against *Escherichia coli*, *Staphylococcus aureus*, and *Bacillus cereus* [203]. Other caffeine–metal nanoparticles have been developed for antibacterial applications. Khan et al. [204] demonstrated the ability of caffeine–gold nanoparticles to inhibit biofilm formation and eliminate mature biofilms. In addition, the nanoparticles showed antibacterial activity against resistant pathogenic bacteria (*Escherichia coli*, *Pseudomonas aeruginosa*, *Staphylococcus aureus*, *Listeria monocytogenes*), demonstrating their potential for treating chronic infections.

Overall, the different types of nanocarriers have shown the potential to improve the therapeutic effect of caffeine. Table 6 provides an overview of the recent research on the development of lipid-, polymer-, and metal-based nanocarriers loading caffeine for biomedical applications.

## 6. Conclusions

Coffee is the most consumed caffeinated beverage, while caffeine can also be found in tea, soft drinks, and energy beverages. Studies on the associations between coffee consumption and a range of health outcomes have been completed. Epidemiological studies reveal that, for the majority of people, coffee consumption is advantageous and adversely connected with risk for a number of diseases. Numerous researchers have recently conducted studies on the effects of caffeine on diseases such as cancer, cardiovascular, immunological, inflammatory, and neurological disorders, among others, as well as in sports, suggesting that this field of study is expanding quickly. To clarify the link between caffeine consumption and specific diseases and to examine consumption patterns in relation to health outcomes, randomized controlled studies are required because association does not imply causality. Because most studies have focused on adults, little is known about the negative consequences of children and adolescents consuming items with caffeine. On the other hand, several advancements in innovative DDS have been made in order to lessen the adverse effects and boost bioavailability for the treatment of various diseases. Thus, DDS have potential importance for clinical applications in several diseases, potentiating the effect of caffeine. However, the growing volume of articles, meta-analyses, and scientific evidence is not yet sufficient to confirm the quality and quantity of caffeine in the treatment of several disorders and in sports, being an avenue to explore in the future.

## Figures and Tables

**Table 1:** Major sources of caffeine and respective levels, according to their usual commercialization volumes/recommend preparations.

Source	Volume (mL)	Caffeine Range (mg)	References
**Coffee**			
Americano coffee	100.0	91.7–213.3	[42]
Decaffeinated coffee	500.0	0.0–13.9	[43]
Instant coffee	125.0	8.7–120.0	[31,41,42,44]
Plain coffee	200.0	68.4–136.9	[42]
Scotland espresso	13.0–90.0	66.0–276.0	[31]
Italy espresso	13.0–31.0	54.0–150.0	[31]
Spain espresso	34.0–104.0	82.0–139.0	[31]
**Tea**			
Black tea	236.0	42.0	[41]
Green tea	236.0	18.0	[41]
Yerba Mate	236.0	40.0	[41]
**Soft drinks**			
Coca-Cola classic	354.0	34.0	[41]
Coca-Cola Energy	354.0	38.0	[41]
Diet Coke	354.0	46.0	[41]
Pepsi	354.0	38.0	[41]
Mountain Dew	354.0	54.0	[41]
Mountain Dew Rise	473.0	180.0	[41]
Ski	354.0	69.0	[41]
Sunkist	354.0	19.0	[41]
**Energy drinks**			
Mountain Dew Amp	473.0	142.0	[41]
Full Throttle	473.0	160.0	[41]
Monster Dragon Tea	680.0	60.0	[41]
Java Monster 300	443.0	300.0	[41]
Red Bull	250.0	80.0	[41]
Rockstar Boom	473.0	160.0	[41]
Rockstar XDurance	473.0	300.0	[41]
**Juice**			
Cran Energy	295.0	70.0	[41]
**Energy shots**			
Bang Shot	88.0	300.0	[41]
5-Hour Energy	57.0	200.0	[41]
TruBrain Extra	29.0	100.0	[41]
Spike Energy Double Shot	125.0	350.0	[41]
**Other beverages**			
Water Joe	591.0	70.0	[41,45]
**Chocolate**			
Dark chocolate	10.0 *	8.0	[41]
Guarana	1.0 *	47.0	[41]

* Values in grams.

**Table 2:** Overview of the latest research regarding caffeine anti-cancer activity.

Target Cancer	Study Type	Model	Caffeine Exposure	Result	Reference
Breast	In vitro	MCF-7 and MDA-MB-231cells	1–10 mM	Caffeine reduced the cell viability in concentrations greater than 2.5 mM for MCF7 and for 5 and 10 mM for MDA-MB-231 cell lines. At the latter concentrations, caffeine induces apoptosis and necrosis in both cell lines.	[57]
Breast	In vitro	MDA-MB-231, MCF7 and MCF10A cells	0.000125 mM	After MDA-MB-231 and MCF7 cells’ treatment with caffeine, there was a change in metabolism towards respiratory-chain phosphorylation with low ratio of free to bound NADH. In combination with cisplatin, there was a decrease in viability and preference of cancer cells over normal breast cells.	[73]
Breast and colon	In vitro	HCT116 and MCF7 cells	0–60 mM	Apoptosis increased in both proliferative and senescent cells after treatment with caffeine at a concentration of 15 mM.	[56]
Carcinoma squamous cells	In vitro	HN5 and KYSE30 cells	0.5–70 mM	Caffeine at concentrations of 20, 50, and 70 mM presented an inhibitory effect and decreased the proliferation rate of both cell lines.	[74]
Endometrial	In vitro	RL95-2, HEC-1-A and KLE cells	0–40 mM	Therapeutic concentration of cisplatin decreased from 4.1 to 1.1 µM and from 163 to 6.6 µM, with caffeine concentrations of 1.1 and 5.3 mM, respectively.	[70]
Glioblastoma multiforme	In vitro	Human GBM and U87-MG cells	1 mM	Pre-treatment of cells with caffeine followed by combined treatment of temozolomide and caffeine significantly decreased cell viability compared to the other groups.	[69]
Glioblastoma multiforme	In vitro	Human GBM, U87MG and T98G 101 cells	0.5–10 mM	In both cell lines, caffeine at 2.5 mM was able to reduce cellular viability, which was more pronounced under hypoxia.	[59]
Lung	In vitro	NCI-H23 and MLC15 cells	0–0.5 mM	After of NCl-H23 cells’ treatment with 0.25 and 0.50 mM caffeine, the size of colonies decreased by 78.1% and 63.9%, respectively. In addition, caffeine induced cell arrest in the G0/G1 phase, reduced the S phase of the cell cycle, and suppressed cell invasion.	[75]
Melanoma	In vitro	Normal human melanocytes COLO829 and C32 cells	100–1000 mM	The results showed the ability of caffeine to reduce the viability of COLO829 and C32 cells by 5–35% and 1–16%, respectively. In addition, it also led to a decrease in thiol degradation and pro-apoptotic effects and did not affect normal melanocytes cells.	[60]
Melanoma	In vitro	B16F10 cells	0.001–0.04 mM	Cells’ pre-treatment with caffeine enhanced the cytotoxic effects induced by dacarbazine. In addition, caffeine increased oxidative stress in a dose-dependent manner.	[58]
Pancreatic ductal adenocarcinoma	In vitro	AsPC-1, BxPC-3, Capan-1, COLO-357, MiaPaCa-2, SU.86.86, PANC-1, and T3M4 pancreatic cancer cells	0.1, 0.2 mM	Caffeine enhanced cell death induced by 5-fluorouracil and gemcitabine, and also decreased the IC_50_ of both chemotherapeutic agents.	[71]
Prostate	In vitro	PC-3 cells	0.5 mM	Caffeine affected cell viability in a dose-dependent manner. Cell migration and invasion ability was more affected by the combination of atorvastatin and caffeine than by caffeine alone. The same was observed for the formation of tumor spheres.	[76]
Glioma	In vitro and in vivo	RT2 cells-induced glioma in male Fischer 344 inbred rat	100 mg/kg/day orally (2 weeks) plus temozolomide given once daily (5 days)	The combination of caffeine with temozolomide inhibited tumor growth compared to the control group.	[68]
Hepatocellular carcinoma	In vitro and in vivo	SMMC-7721 and Hep3 cell lines and Male BALB/c nude mice	0–32 mM (in vitro) 20 mg/kg/day injected IP every other day for (2 weeks)	Caffeine decreased the viability of both cell lines and had a synergistic effect with 5-fluorouracil. In addition, tumor growth was suppressed, and tumor weight was reduced in mice treated with caffeine alone or in combination with 5-fluorouracil.	[77]
Osteosarcoma, fibrosarcoma	In vitro and in vivo	HOS, HT1080 and LM8 cells and athymic nude mice	0.5 mM (in vitro) 100 mg/kg injected IP on days 2 to 4 to the treatment (1 week). The treatment was performed two times.	The combination of cisplatin and caffeine decreased cell viability compared with cisplatin alone. In vivo, after implantation of LM8 and HT1080 cells, the combination of cisplatin + caffeine decreased tumor volume and weight.	[78]
Pleomorphic rhabdomyosarcoma	In vitro and in vivo	RMS cells, Athymic nu/nu nude mice	0.5 and 1 mM (in vitro) 100 mg/kg/day injected IP daily (3 weeks)	Caffeine showed the ability to enhance the antiproliferative effects of valproic acid. In vivo, the group treated with caffeine and valproic acid showed a reduction in tumor volume compared to the control group. This was also confirmed in the group treated with *Salmonella typhimurium* A1 receptor in combination with caffeine and valproic acid.	[79]
Renal cell carcinoma	In silico, in vitro, and in vivo	ACHN and 786-O cells, and BALB/c nude mice	0–0.016 mM intragastrically administered for 34 consecutive days	The molecular docking studies demonstrated that caffeine was able to bind to G6PDH at the NADP+ binding site, which is a biomarker and potential therapeutic target for renal cell carcinoma. In addition, caffeine was able to decrease the viability and proliferation of both cell lines and in the in vivo studies.	[64]
Colorectal	In vivo and in silico	Swiss Webster mice	50 mg/kg/day, intragastrically 5 times a week (10 weeks)	Mice treated with caffeine alone or in combination with chlorogenic acid decreased the expression of IL-6, IL-17, and TNF-α.	[80]
Fibrosarcoma	In vivo	Adult albino mice	1.030, 2.060 and 4.120 mM in drinking water administered daily (8 weeks)	In caffeine-treated mice, tumor incidence, size, and growth rate decreased with the increase in caffeine concentration. In addition, caffeine-treated mice had a higher percentage of cytotoxic T cells and higher TNF-α and IFN-γ levels.	[61]
Fibrosarcoma	In vivo	Adult Syrian golden hamsters	100 mg/kg/day, intragastrical administration; treatment started 3 days before inoculation with sarcoma cells and continued for 14 days	Administration of metformin and caffeine resulted in inhibition of fibrosarcoma growth.	[81]
Melanoma	In vivo	Albino mice and C57BL/6J mice	4.120 mM daily in drinking water (3 or 6 weeks)	In the carcinogen-induced tumor model, the groups treated with caffeine alone decreased the tumor growth rate from 5.3 mm^2^/day to 2.6 mm^2^/day. The combination with anti-PD1 led to a more pronounced decrease (0.9 mm^2^/day).	[82]
Osteosarcoma	In vivo	Athymic nu/nu nude mice	100 kg/kg/day, orally administered for 14 consecutive days	The osteosarcoma mice model (patient-derived orthotopic xenograft) treated with cisplatinum + oral recombinant methioninase + caffeine, showed the most marked decrease in comparison to the other groups.	[83]
Synovial sarcoma	In vivo	Athymic nu/nu nude mice	100 mg/kg/day, orally administered for 14 consecutive days	The combination of oral recombinant methioninase and caffeine reduced tumor volume.	[72]

IC_50_, half-maximal inhibitory concentration; NADH, nicotinamide adenine dinucleotide; MTT assay, (3-[4,5-dimethylthiazol-2-yl]-2,5 diphenyl tetrazolium bromide) assay; Anti-PD1, anti-Programmed Cell Death Protein 1; TNF-α, tumor necrosis factor alpha; IFN-γ, interferon gamma; G6PDH, glucose-6-phosphate dehydrogenase; NADP+, nicotinamide adenine dinucleotide phosphate; IP, intraperitoneally.

**Table 3:** Overview of the latest research regarding caffeine anti-inflammatory activity and impact on the immune system.

Target/Disease	Study Type	Model	Caffeine Exposure	Result	Reference
Anti-inflammatory effect and immunomodulation	In vitro	Human peripheral blood mononuclear cells	1.16 mM	Caffeine reduced the levels of several cytokines (IL-8, MIP-1*β*, IL-6, IFN-*γ*, GM-CSF, TNF-*α*, IL-2, IL-4, MCP-1, and IL-10. It also inhibited STAT1 signaling.	[109]
Bronchopulmonary dysplasia	In vitro	THP-1-derived macrophages	100–800 μM	There was a decrease in NLRP3 inflammasome activation, ASC speck formation, and caspase 1 cleavage. In addition, IL-1β and IL-18 secretion decreased, as well as the phosphorylation of MAPK and NF-kB pathway members.	[110]
Immunomodulation	In vitro	Monocytes and macrophage	300–1000 µM	Caffeine suppressed TNF-α and Akt signaling in both LPS-activated macrophage subtypes, inhibited STAT/IL-10 signaling in macrophage colony-stimulating factor, and significantly increased the expression of A2a and downregulated mTOR phosphorylation in M-macrophages.	[111]
Immunomodulation	In vitro	Mesenchymal stem cells and neutrophiles	0.1–1 mM	Caffeine-treated mesenchymal stem cells produced fewer reactive oxygen species and increased phagocytosis of neutrophils co-cultured with mesenchymal stem cells.	[112]
Immunomodulation	In vitro	Mesenchymal stem cells and neutrophiles	0.1–1 mM	Caffeine treatment increased the viability of co-cultured neutrophils.	[113]
Melanoma	In vitro and in silico	Mel1 and Mel3 cells	1 and 2 mM	After caffeine treatment, there was a decrease in the levels of IL-1β, IP-10, macrophage inflammatory protein 1-α, and CCL4. On the other hand, the expression of regulated and normal T cells decreased in the Mel3 cell line.	[114]
Autoimmune encephalomyelitis	In vitro and in vivo	Primary microglia and BV2 cells C57BL/6 mice were immunized to induce autoimmune encephalomyelitis	2 mM (in vitro) 10, 20 and 30 mg/kg/day in drinking water (30 days) after immunization with MOG_35–55_	Caffeine decreased clinical score, inflammatory cell infiltration degree of the demyelination, and microglia stimulation in mice. In addition, it increased LC3-II/LC3-I levels and decreased NLRP3 and P62 levels.	[95]
Choroidal neovascularization	In vitro and in vivo	Laser photocoagulation C57BL/6j mice model	200, 400 µM (in vitro); before laser photocoagulation (day 9): 20 mg/kg at day 0 and 10 mg/kg at day 1–4 and day 7 to 8; after laser photocoagulation: 10 mg/kg for 2 weeks (excluding weekends)	Significantly reduced the migration of retinal and choroidal endothelial cells (in vitro). Decreased choroidal neovascularization and inflammatory (mononuclear phagocytes) cells recruitment to the lesion area.	[97]
Depression	In vitro and in vivo	CBA × C57BL/6 F1 mice and syngeneic splenocytes	Transplantation (IV injection) with 15 × 10^6^ splenocytes previously treated with 100 µg of caffeine for 25 min	Immune cells treated with caffeine and transplanted into depressive-like mice resulted in an increase in neuronal density and anti-inflammatory cytokines (IL-10 and IL-4) and a decrease in proinflammatory cytokines (IL-1β, INF-γ, and TNF-α).	[115]
Infection	In vitro and in vivo	Peritoneal macrophages and Swiss mice infected with *L. Monocytogenes*	0.0257–25.7 μM (in vitro) 0.05, 0.5, 5 mg/Kg of caffeine IV injected 30 min after mice infection	In mice, the leucocyte infiltration in the peritoneal cavity decreased after caffeine treatment. In addition, mRNA expression of IL-1β, IL-6, and the enzyme inducible nitric oxide synthase were decreased, whereas IL-10 was increased.	[116]
Immunological and metabolic anomalies in obesity	In vitro and in vivo	Male Sprague-Dawley rat, RAW 264.7 macrophage and HepG2 cells	50, 100, 150 mΜ (in vitro*)* High-fat-diet (6 weeks) induced hepatic steatosis mice were treated with 20 mg/kg/day by oral gavage (6 weeks)	In caffeine-treated mice, the profiles of TNF−α, MCP-1, IL-6, intercellular adhesion molecule, and nitrite were suppressed. In addition, live white adipose tissue and muscle macrophages and their cytokine levels also decreased.	[117]
Retinal inflammation	In vitro and in vivo	Ischemia reperfusion (I/R) injury mice model	1–100 µM (in vitro); 10 µL at 97.8 mM instilled 60 min before and after I/R reperfusion, twice a day for 72 h	Caffeine reduced the secretion of IL-1β, IL-6, and TNF-α and restored the integrity of retinal cell monolayer (in vitro). Instilled caffeine reduced IL-6 mRNA levels and maintained BDNF physiological levels in the retina.	[100]
Rheumatoid arthritis	In vitro and in vivo	Mesenchymal stem cells and Wistar rats	0–1 mM (in vitro); 14 days after rheumatoid arthritis induction, mice were injected IP with 2 × 10^6^ cells previously treated with 0.5 mM caffeine for 48 h	Caffeine at a concentration of 0.5 mM promoted lower levels of cytokines, such as IFN-γ, IL-6, and IL-1β, and higher levels of IDO and TGF-β. In addition, cells treated with caffeine diminished the severity of rheumatoid arthritis in vivo and caused a decrease in serum levels of C-reactive protein, nitric oxide, myeloperoxidase, and TNF-α.	[96]
Cognitive impairment	In vivo	BALB/c mice	0.025, 0.05, 0.1 mg of caffeine intranasally administered (10 µL) 1 day before ischemia-induced cognitive impairment in mice, and the next 7 consecutive days	Caffeine improved the behavior outcomes of ischemic mice and reduced the expression of proinflammatory biomarkers (TNF-α, IL-6) and increased the levels of anti-inflammatory cytokines (IL-10).	[118]
Hepatic fibrosis—antioxidant and anti-inflammatory	In vivo	Hepatic fibrosis Sprague Dawley rats	50 mg/kg/day orally administered (8 weeks)	Decreased fibrosis and necro-inflammation; decreased LPAR1, TGF-β1, CTGF, α-SMA, and LPAR1 expression; improved liver function.	[119]
Hydrocephalus	In vivo	Kaolin-induced hydrocephalus mice neonates	50 mg/kg/day of caffeine were administered to dams by gavage or water (21 days) and lactated the neonates	Administration of caffeine to dams reduced cell death and increased the neurons dendritic arborization in the sensorimotor cortex and striatum of the mice neonates and improved hydrocephalic deficits and behavioral development.	[120]
Immunomodulation and anti-inflammatory effect	In vivo	Nile tilapia	Diet containing 5 and 8% *w*/*w* (21 days)	Caffeine supplemented diet prevented alterations caused by hypoxia, such as ATP hydrolysis and consequent accumulation in the extracellular environment.	[121]
Inflammation and adenosinergic system in cerebellum	In vivo	Ethanol-induced inflammation in Wistar and UChB rats	15.4 mM/day in 10% ethanol solution (55 days)	Caffeine reduced gene expression of A1 and A2a receptors and increased and reduced A1 and A2a protein levels, respectively, in the cerebellum. Caffeine also attenuated the inflammation, demonstrating a neuroprotective role.	[122]
Neuroinflammation	In vivo	Sprague Dawley rats	60 mg/kg/day administered orally by gavage (2 days)	Caffeine/modafinil increased the levels of anti-inflammatory (IL-4 and IL-10) and decreased proinflammatory (TNF-α, IL-1β) cytokines in the hippocampus. Treatment decreased microglial immunoreactivity and improved inflammatory response and anxious behavior.	[123]
Neurotoxicity	In vivo	Tramadol-induced damage in cerebellum rat model	37.5 mg/kg/day administered orally by gavage (21 days)	Caffeine upregulated autophagy-related genes and reduced the expression of inflammatory and apoptosis markers, demonstrating neuroprotective effects in the cerebellum.	[124]
Neurotoxicity—antioxidant and anti-inflammatory	In vivo	Albino rats	20 mg/kg/day IP injected (30 days)	Caffeine reduced oxidative stress and restored TNF-α levels in cerebral tissues.	[125]
Oxygen-induced inflammatory lung injury	In vivo	Neonatal rats	10 mg/kg IP injected every 48h (15 days)	Under hyperoxia, caffeine decreased pro-inflammatory mediators (TNF-α, IL-1α, IL-1β, IFN-γ) and NF-kB, and decreased infiltrating cells in the lung. Opposite effects were observed in normoxiaconditions.	[108]
Dental pain	Clinical Trial	Patients with acute postoperative dental pain	100 mg (single dose)	Caffeine improved the effect of ibuprofen in the treatment of moderate postoperative dental pain.	[126]

IL, interleukin; TNF-*α*, tumor necrosis factor alpha; IFN-*γ*, interferon gamma; MCP-1 (monocyte chemoattractant protein-1); STAT1, Signal Transducer and Activator of Transcription 1; Akt, protein kinase B; AMPK, adenosine monophosphate-activated protein kinase; mTOR, mammalian target of rapamycin; NLRP3, NLR family pyrin-domain-containing 3; NF-*k*B, nuclear factor-*κ*B MAPK, mitogen-activated protein kinase; IP-10, interferon gamma-induced protein 10; CCL4, CC motif chemokine ligand 4; TGF-*β*, transforming growth factor beta; CTGF, connective tissue growth factor; *α*-SMA, alpha smooth muscle actin; LPAR1, lysophosphatidic acid receptor 1; LPS, lipopolysaccharide; M-MFs, inflammation-resolving macrophages; GM-MFs, inflammation-promoting macrophages; NF*κ*B1, nuclear factor kappa B subunit 1; HMGB1, high mobility group box 1 protein; BDNF, brain-derived growth factor.

**Table 4:** Overview of the latest research regarding caffeine in neurodegenerative diseases.

Disease	Study Type	Model	Caffeine Exposure	Result	Reference
Parkinson’s	In silico	Molecular docking simulations	N/A	Caffeine was able to bind at position 28 in both wild-type and mutant parkin proteins.	[139]
Alzheimer’s	In silico	Molecular docking simulations	N/A	In the presence of caffeine, the distances between the inter-residual increased, leading to the breakdown of hydrophobic contacts, ultimately destabilizing the Aβ protofibrils.	[144]
Parkinson’s	In vitro	Transgenic *Caenorhabditis elegans*	10 mM	Caffeine was able to prevent neuronal cell loss in 96% of dopaminergic neurons.	[138]
Alzheimer’s	In vitro	SHSY5Y cells	0.6 and 1 mM	Both concentrations were able to reduce beta-amyloid neurotoxicity.	[145]
Alzheimer’s	In vitro	SH-SY5Y wild-type and N2a cells	100 µM	In the presence of caffeine, the level of ADAM10 protein increased to 138.5 ± 9.2%, and the levels of APP protein level and ROS decreased to 85.4 ± 3.6% and 48.8 ± 3.2%, respectively.	[146]
Alzheimer’s	In vitro	HEK293 cells	0.1–10 mM	Caffeine induces conformational changes in muscle nicotinic acetylcholine receptors, which are molecular targets of Alzheimer’s disease.	[147]
Synaptic transmission and plasticity	In vitro	Dorsal hippocampus slices of C57bl\6j mice and A2aR knockout mice	50 μM	Caffeine increased synaptic transmission by 40%, decreased facilitation of paired pulse, and decreased the amplitude of long-term potentiation by 35%.	[15]
Cd-induced neurodegeneration	In vitro and in vivo	HT-22 and BV-2 cells and wild-type C57BL/6N male mice	30 mg/kg/day IP injected (2 weeks)	Caffeine reduced ROS, lipid peroxidation and 8-dihydro-8-oxoguanine levels. It also attenuated neuronal loss, synaptic dysfunction, and learning and cognitive deficits.	[148]
Parkinson’s	In vivo	Swiss mice and Wistar rats	31.2 mg/kg given orally by gavage	Caffeine administration reduced the catalepsy index and increased the number of ipsilateral rotations.	[149]
Hypoxic ischemia	In vivo	Sprague Dawley mice	1.5 mM in drinking water until 16 postnatal days	Pre-treatment with caffeine reduced brain infarct after hypoxia ischemia and also restored brain activity.	[150]
Acetaminophen-induced neurotoxicity	In vivo	Swiss albino mice	20 mg/kg IP injected 30 min after treatment with acetaminophen	Treatment with caffeine and acetaminophen reduced the formation of ROS compared with the acetaminophen group. In addition, the survival time of caffeine-treated mice increased by 33%.	[151]
Parkinson’s	In vivo	C57BL/6 mice with motor behavioral deficit induced by 1-methyl-4-phenyl-1,2,3,6-tetrahydropyridine	20 mg/kg/day, 7 days before MPTP-induced neurodegeneration and 7 days after	Caffeine improved behavioral and neurotransmitter recovery against the induced toxicity. It was also able to restore antioxidant levels and suppress neuroinflammation.	[152]
Hypoxic ischemia	In vivo	Wild-type C57/bl6 specific pathogen-free mice	5 mg/kg IP injected (120 days)	Caffeine administration after hypoxic ischemic brain injury reduced lesions in the gray and white matter and the number of amoeboid microglia and apoptotic cells. The expression of pro-inflammatory cytokines also decreased.	[153]
Apnea of prematurity	In vivo	Infection-free pregnant Sprague Dawley rats	20 mg/kg 1 day followed by 5 mg/kg/day over 14 days or 80 mg/kg 1 day followed by 20 mg/kg/day over 14 days, IP injected	Caffeine administration in normoxia reduced oxidative stress and hypermyelination, and increased Golgi bodies. Caffeine at standard and high doses could provide neuroprotective effects.	[154]
Parkinson’s	In vivo	C57BL/6 male mice	5.1 mM in drinking water	Caffeine protected against synucleinopathy by modulating α-syn-induced apoptosis, microglial, and astrocytic activation in the striatum.	[142]
Neuroprotection	In vivo	Male Swiss mice	1.5 mM in drinking water (4 weeks)	The number of A2a receptors was decreased in the hippocampus of mice that consumed caffeine. The aged mice treated with caffeine presented more pyknotic neurons in the hippocampus and reduced damage.	[155]
LPS-induced oxidative stress and neuroinflammation	In vivo	C57BL/6N male mice	3 mg/kg/day IP injected (6 weeks)	The LPS-injected group had enhanced expression of Bax and caspase-3. On the other hand, these markers were reduced in the group treated with caffeine, and this treatment also caused a restoration of the synaptic markers.	[156]
Diabetes	In vivo	Male GK and Wistar–Hannover–Galas rats	5.1 mM in drinking water (4 months)	Caffeine prevented the GFAP, vimentin, and SNAP25 alterations caused by diabetes, and also improved memory deficits.	[157]
Alzheimer’s	In vivo	Wild-type N2 and CL2006 worms	Worms were cultured in 200 and 400 μM caffeine-treated plates	The treatment prevented amyloid beta-peptide paralysis, decreased acetylcholinesterase activity, and decreased amyloid beta-peptide mRNA levels.	[158]
Parkinson’s	In vivo	C57BL/6J mice	50 mg/kg/day in drinking water	The co-administration of caffeine and eicosanoyl-5-hydroxytryptamide resulted in decreased accumulation of phosphorylated α-synuclein, maintenance of neuronal integrity and function, reduction in neuroinflammation, and improvement in behavioral performance.	[143]
Parkinson’s	Clinical trial	Parkinson’s disease patients	100 mg (single dose)	Caffeine treatment reduced the number of errors in patients and controls on the Stroop and Choice reaction time and enhanced dual item accuracy on the rapid visual serial presentation task.	[159]

ADAM10, A disintegrin and metalloproteinase domain-containing protein 10; APP, amyloid-beta precursor protein; ROS, reactive oxygen species; LPS, lipopolysaccharides; GFAP, glial fibrillary acidic protein; SNAP25, synaptosomal-associated protein, 25kDa; N/D, non-disclosed; MPTP, 1-methyl-4-phenyl-1,2,3,6-tetrahydropyridine; N/A, not applicable.

**Table 5:** Overview of the latest research regarding caffeine’s impact on cardiovascular diseases.

Study Type	Model	Result	Reference
Systematic review	Review of prospective studies	Regular and moderate coffee consumption (1–2 cups/day) is not associated with hypertension risk. Higher coffee consumption has a protective effect.	[168]
Prospective	347,077 volunteers (37–73 years old, UK Biobank)	Coffee consumption may lead to a slight increase in CVD risk.	[162]
Prospective	2278 volunteers (18–80 years old)	Caffeine metabolites are responsible for lowering the risk of hypertension.	[169]
Prospective	20,487 (35–94 years old)	Moderate coffee consumption (3–4 cups/day) has been associated with lower CVD mortality.	[164]
Prospective	>500,000 individuals (40–69 years old)	The consumption of 2–3 cups of coffee per day (121–182 mg caffeine/day) was associated with a low risk of coronary artery disease.	[165]
Prospective	23,878 individuals (>20 years old)	Higher caffeine intake (>100 mg/day) was associated with lower CVD mortality.	[166]
Prospective	362,571 individuals (37–73 years old, UK Biobank)	High coffee consumption (>6 cups/day) increases levels of low-density lipoprotein cholesterol, total cholesterol, and apolipoprotein B, thereby increasing the risk for CVD.	[163]
Prospective	1095 individuals (mean age 53 ± 14 years old)	Moderate coffee consumption (>3 cups/day) reduces CVD risk factors such as arterial stiffness and high blood pressure	[167]
Randomized Controlled Trial	12 volunteers (19–39 years old)	Administration of caffeine (200 mg, 12 h intervals) during sleep deprivation reduced HR and increased HF-HRV. The concentration effect was nonlinear. No significant interaction between sleep deprivation and caffeine intake	[170]
In vitro in vivo	Primary human and mouse aortic VSMCs, immortalized mouse aortic VSMCs; restenosis mice model (apoe−/−C57BL/6 J)	In vitro, caffeine (2 mM) induced autophagy by inhibiting mTOR signaling and decreased proliferation of VMCs by inhibiting WNT signaling. In vivo, caffeine at 2.57 mM (in drinking water, 2 weeks before and after injury) decreased vascular restenosis.	[171]
In vivo	Zebrafish	Caffeine (128 and 334 µM in zebrafish culture water) caused a similar decrease in HR.	[172]

HR, heart rate; HF-HRV, heart rate variability; mTOR, mammalian target of rapamycin; VSMCs, vascular smooth muscle cells.

**Table 6:** Overview of the latest research regarding nanocarriers loading caffeine for biomedical applications.

Nanosystem	Method	Composition	Application	Model	Result	Reference
**Lipid-based nanosystems**						
Liposomes	Thin-film hydration	Lecithin, polysorbate 80, polysorbate 20	Alopecia	Wistar rats	Improves skin delivery, weight, and hair length.	[198]
Liposomes	Thin film hydration	Phospholipid, cholesterol	Skin drug delivery	Abdominal skin of WBN/ILA-Ht hairless rats	DPPG liposomes enhanced skin penetration by disrupting the lipidic barrier of stratum corneum.	[194]
Liposomes	High-pressure homogenization	Phosphatidylcholine, propylene glycol	Skin drug delivery	Full-thickness abdominal human skin	Propylene glycol increased liposome deformability and improved skin permeation of caffeine.	[196]
Lipidic nanosystems	High-pressure homogenization	Trilaurin, oleic acid, pluronic F68, imiquimod	Cancer	Orthotopic breast cancer mice model	Caffeine slightly improved antitumor activity.	[201]
Lipid nanocapsules	Phase inversion temperature	Miglyol 812 N, Kolliphor HS 15, Phospholipon 90G	Skin drug delivery	Porcine skin	Caffeine was not successfully encapsulated. Nanocapsules improved the transdermal permeation of caffeine.	[195]
Semi-solid nanostructured lipid carriers	Two-stage homogenization method, high shear homogenization, ultrasonication	Compritol^®^ 888 ATO and Precirol^®^ ATO 5, argan oil, Poloxamer 407	Cosmetics, skin drug delivery	Wistar rat full-thickness dorsal skin	NLCs exhibited a high capacity for deposition and permeation through the skin.	[197]
Proniosomes	Coacervation phase separation	Cholesterol, span 60, lecithin	Brain delivery—migraine	Swiss albino mouse abdominal skin and albino rabbit ear	Increased caffeine permeation through the skin and caffeine levels in blood and brain compared to orally administered caffeine. No evidence of skin irritation.	[188]
Nanoemulsions	Low energy emulsification	Dicaprylyl ether, ethylhexyl isononanoate, potassium lauroyl wheat amino acids, palm glycerides and capryloyl glycine	Cosmetics, skin drug delivery	Abdominal human epidermis	Did not improve skin permeation of caffeine compared to emulsion.	[205]
Nanoemulsions	Low energy emulsification	Volpo-N10, oleic acid or eucalyptol	Skin drug delivery	Human full-thickness skin	Increased permeation and retention of caffeine in hair follicles and skin.	[200]
Pickering emulsions stabilized by magnesium oxide NPs	High shear homogenization	Wheat germ oil, magnesium oxide NPs	Oral drug delivery—hepatoprotective	Wistar rats intoxicated with CCl4	Decreased proliferation of cancer cells, moderate reduction in oxidative stress and inflammatory markers, similar to caffeine solution. Increased catalase levels compared to caffeine.	[206]
**Polymer-based nanosystems**						
Polymeric nanoparticles	Emulsion polymerization	Methyl methacrylate, CTAB or sodium dodecyl sulfate	Antifungal	*C. albicans*	CTAB–caffeine nanoparticles inhibited the growth of *C. albicans*.	[207]
Polymeric nanoparticles	Desolvation	Gelatin	Cancer	B16F10, L929 cell lines	Inhibited the proliferation of murine melanoma cells (B16F10) and induced apoptosis without causing cytotoxic effects on normal fibroblast cells (L929).	[202]
**Metal-based nanosystems**						
Silver complexes anchored to magnetic NPs	Covalent conjugation and complexation	Chloro-functionalized Fe3O4 magnetic NPs, caffeine N-heterocyclic carbene-silver complex	Cancer	HepG2, WRL-68 cell lines; *E. coli*, *P. aeruginosa*, *S. aureus*, *L. monocytogenes*	Enhanced cytotoxic effects against HepG2 cells and antibacterial activity against *E. coli*, *S. aureus* and *B. cereus*. Hyperthermia studies showed that the nanosystems reached a temperature of 47 °C, which is suitable for anticancer applications	[203]
Silver nanoparticles	Chemical reduction	Silver nitrate, gallic acid, (-)-epicatechin-3-gallate or caffeine	Cancer	B16-F0, COLO 679 cell lines	EGCG- and caffeine-stabilized AgNPs were the most and less effective against the tested cancer cell lines.	[208]
Gold nanoparticles	Chemical reduction	Gold (III) chloride trihydrate	Antibacterial	*E. coli*, *P. aeruginosa*, *S. aureus*, *L. monocytogenes*	Inhibition of biofilm formation and removal of mature biofilms. Antibacterial activity against resistant pathogenic bacteria.	[204]
**Crystal-based nanosystems**						
Nanocrystals	Pearl-milling	Carbopol^®^ 981, propylene glycol	Skin drug delivery	Human volunteers, arm skin	Nanocrystals with a size of 694 nm showed a delayed, but higher and longer delivery of caffeine, being detected in serum for at least 5 days.	[209]

NLCs, nanostructured lipid carriers; AgNPs, silver nanoparticles; DPPG, 1,2-dipalmitoyl-sn-glycero-3-phosphoglycerol, sodium salt; CTAB, cetyltrimethylammonium bromide; EGCG, (-)-epicatechin-3-gallate.

## Data Availability

Data sharing not applicable.
